# Perceived parental phubbing and Internet gaming disorder symptoms among adolescents: the mediating roles of fear of missing out and self-control

**DOI:** 10.3389/fpsyg.2026.1906219

**Published:** 2026-07-31

**Authors:** Cancan Li, Yali Zhang, Yuhong Ren

**Affiliations:** 1 Department of Psychology, Hebei Normal University, Shijiazhuang, China; 2 Department of Foreign Language Teaching and Research, Hebei Normal University, Shijiazhuang, China

**Keywords:** adolescents, fear of missing out, Internet gaming disorder symptoms, perceived parental phubbing, self-control

## Abstract

**Objective:**

To examine the association between perceived parental phubbing and Internet gaming disorder symptoms among adolescents, as well as the mediating roles of fear of missing out and self-control in this association.

**Methods:**

A questionnaire survey was conducted among 5,476 adolescents using the Perceived Parental Phubbing Scale, the Fear of Missing Out Scale, the Self-Control Scale, and the Internet Gaming Disorder Symptoms Scale.

**Results:**

Perceived parental phubbing was significantly and positively correlated with adolescents’ Internet gaming disorder symptoms. The mediating effect of fear of missing out between perceived parental phubbing and Internet gaming disorder symptoms was significant, and the mediating effect of self-control between perceived parental phubbing and Internet gaming disorder symptoms was also significant. The serial mediation pathway of perceived parental phubbing → fear of missing out → self-control → Internet gaming disorder symptoms was significant, although the effect size was relatively small.

**Conclusion:**

Adolescents’ Internet gaming disorder symptoms are closely associated with their perceived parental phubbing, fear of missing out, and level of self-control. In the digital-intelligence era, prevention of adolescent Internet gaming disorder symptoms may begin with improving the family digital-interaction environment, alleviating fear of missing out, and cultivating self-control.

## 1 Introduction

Internet gaming disorder symptoms refer to persistent and recurrent gaming behavior accompanied by physiological, psychological, and social functional impairment; the relevant diagnostic condition has been incorporated into the classification systems for mental disorders in both the DSM-5 and ICD-11 (Gursesli et al., 2025). Studies have shown that the prevalence of Internet gaming disorder symptoms among adolescents is approximately 6.70 to 12% and has shown an increasing trend year by year (Liao et al., 2022; Zhou et al., 2024). Internet gaming disorder symptoms may impair individuals’ neurophysiological functioning, reduce life satisfaction, and induce problems such as emotional distress and substance abuse (Amendola et al., 2025; Coutelle et al., 2024; Chang and Lee, 2024). More importantly, Internet gaming disorder symptoms may not only endanger adolescents’ current physical and mental health, but their negative effects may also extend into adulthood. Therefore, prevention of adolescent Internet gaming disorder symptoms is of critical importance. Existing research has examined the influence of family factors such as parenting styles, attachment patterns, and family support on adolescent Internet gaming disorder symptoms (Novak et al., 2026; Petrescu et al., 2025), but little attention has been paid to perceived parental phubbing. It is therefore necessary to analyze the effect of perceived parental phubbing on adolescent Internet gaming disorder symptoms and the underlying mechanisms. According to the I-PACE model (Interaction of Person-Affect-Cognition-Execution model) (Brand et al., 2019), problematic Internet use is the product of dynamic interactions among individual susceptibility traits, affective responses, cognitive processing, and executive functioning in specific contexts. Therefore, the present study includes fear of missing out (an affective response) and self-control (executive functioning) to examine their mediating and serial mediating roles.

### 1.1 Perceived parental phubbing and Internet gaming disorder symptoms

Perceived parental phubbing refers to parents’ behavior of neglecting children’s needs and interrupting parent–child interactions because they focus on their mobile phones while spending time with or communicating with their children (Lin et al., 2025). The Compensatory Internet Use Theory (CIUT) holds that when individuals’ psychological needs in the real world are frustrated, they turn to cyberspace to seek substitute satisfaction (Kardefelt-Winther, 2014). Parents’ persistent ‘head-down’ phone use may be associated with lower-quality parent–child interaction, weaker emotional bonds, and higher levels of social neglect, making it difficult for adolescents’ basic relational needs to be satisfied (Yin et al., 2024). The immersive virtual world of online games can provide immediate feelings of achievement, control, and community belonging (Pirrone et al., 2024), and can easily become a pathway through which adolescents compensate for real-life emotional deprivation and seek psychological satisfaction, thereby being associated with more severe Internet gaming disorder symptoms. Existing research has shown that perceived parental phubbing is positively correlated with adolescents’ problematic Internet use (Liu et al., 2024). Accordingly, this study proposes Hypothesis 1: perceived parental phubbing can significantly and positively predict adolescent Internet gaming disorder symptoms.

### 1.2 The mediating role of fear of missing out

Fear of missing out (FoMO) refers to a diffuse, complex emotional experience dominated by anxiety and accompanied by negative feelings such as fear, loss, worry, and frustration arising from the possibility of missing important information or novel events (Zhang et al., 2021). Parents’ excessive attention to mobile phones and neglect of their children may weaken the quality of parent–child interaction, be associated with adolescents’ heightened experiences of rejection and neglect, and hinder the establishment of secure parent–child attachment relationships (Bowlby, 1969; Wang et al., 2026). To alleviate negative emotions such as feelings of exclusion, adolescents are highly likely to overattend to peer acceptance in order to confirm their sense of belonging. Such concerns about being disconnected from social networks are significantly and positively correlated with individuals’ fear of missing out (Geng et al., 2021; Yin et al., 2024). In addition, General Strain Theory (GST) holds that individuals who encounter stressful events develop negative emotional experiences, which in turn give rise to problematic behaviors (Agnew, 1992). As a negative emotion, fear of missing out may trap adolescents in an anxious state and produce psychological distress (Ding et al., 2026). Fear of missing out (FoMO), as a negative emotion, can lead adolescents into anxiety and psychological distress (Ding et al., 2026). Although social media use can provide temporary emotional distraction, the relative homogeneity and uniformity of its content often fail to truly satisfy adolescents’ needs for group belonging and deep connections. Consequently, it fails to fundamentally alleviate FoMO, and may even amplify negative emotions through social comparison and information overload (Burnell et al., 2019; Nowland, 2026). In contrast, online games alleviate this anxiety by enhancing adolescents’ sense of belonging with peers and providing timely feedback and rewards, which is associated with higher levels of Internet gaming disorder symptoms (Caba-Machado et al., 2024). Research has found a significant positive correlation between fear of missing out and Internet gaming disorder symptoms (Akbari et al., 2021). Similar studies also indicate that fear of missing out mediates the associations of childhood neglect and poor parent–child relationships with problematic Internet use (Koca and Saatçı, 2022; Zhang et al., 2023). Accordingly, Hypothesis 2 is proposed: fear of missing out mediates the association between perceived parental phubbing and adolescent Internet gaming disorder symptoms.

### 1.3 The mediating role of self-control

Self-control refers to an individual’s ability to inhibit responses and choose delayed gratification in the face of immediate temptation or impulses in order to achieve long-term goals (Baumeister et al., 2007). According to the Strength Model of Self-Control, when limited self-control resources are occupied by environmental factors and negative emotional states, individuals’ self-control ability is weakened (Baumeister et al., 2007). Perceived parental phubbing is closely associated with adolescents’ negative emotions such as feeling neglected and rejected; individuals need to consume limited psychological resources to alleviate these negative emotions, which weakens their self-control function. Empirical studies have shown that perceived parental phubbing is significantly and negatively correlated with self-control (Jiang et al., 2023), and individuals whose self-control resources are impaired have more difficulty suppressing their pursuit of immediate rewards and are at higher risk of Internet gaming disorder symptoms (Gong et al., 2022; Müller et al., 2025). In addition, according to the I-PACE model, executive functioning is a key variable influencing problematic Internet use (Brand et al., 2019). As a core component of executive functioning, self-control plays a key inhibitory role when individuals face the temptation of online games (Hu et al., 2025). Adolescents’ brain development is not yet mature, and their self-control ability is limited. In the digital-intelligence era, the immediate feedback and convenient social connection provided by online games make it difficult for adolescents to inhibit gaming temptations, increasing the risk of Internet gaming disorder symptoms (Rochat et al., 2026). Moreover, empirical research has shown that self-control is a protective factor against many forms of problematic behavior (Hu et al., 2025; Lei et al., 2020; Vazsonyi et al., 2025), and that self-control mediates the association between parent–child conflict and problematic Internet use (Bağatarhan et al., 2023). Accordingly, Hypothesis 3 is proposed: self-control mediates the association between perceived parental phubbing and adolescent Internet gaming disorder symptoms.

### 1.4 The serial mediating roles of fear of missing out and self-control

Ego Depletion Theory (EDT) holds that self-related activities, such as self-control and emotion regulation, consume individuals’ limited psychological energy (Baumeister, 2003; Ding et al., 2024). Individuals with high fear of missing out need to expend substantial psychological resources to regulate the irritability and unease caused by concerns about missing out; prolonged emotional depletion is likely to induce ego depletion and thereby weaken self-control ability (Xu and Tian, 2023). Empirical findings indicate a significant negative correlation between fear of missing out and self-control (Ding et al., 2026; Jiao and Cui, 2024). Second, the I-PACE model suggests that addictive behavior results from dynamic interactions among core personal characteristics, affective responses, cognitive responses, and executive functioning (Brand et al., 2019). Perceived parental phubbing, as a negative parent–child interaction and an individual risk factor, may lead adolescents to experience being neglected and rejected, impede the satisfaction of their belongingness needs, and increase their fear of missing out (Wang et al., 2026). This sustained state of anxiety consumes psychological resources and weakens executive functions such as self-control, making it difficult for individuals to suppress their impulse to go online and thereby increasing Internet gaming disorder symptoms (Liu et al., 2025). In summary, Hypothesis 4 is proposed: fear of missing out and self-control may play a serial mediating role between perceived parental phubbing and adolescent Internet gaming disorder symptoms.

### 1.5 Current study

The relationship between perceived parental phubbing and adolescent Internet gaming disorder symptoms has attracted broad scholarly attention. Existing research has mainly focused on smartphone addiction or social media dependence (Geng et al., 2021; Niu et al., 2020; Yin et al., 2024), while Internet gaming disorder symptoms has rarely been examined specifically. The emotional deficits caused by perceived parental phubbing may be more easily compensated through the immersive experience unique to games, making it necessary to examine it as an independent outcome variable. Moreover, previous studies have mostly examined the mediating roles of fear of missing out or self-control separately (Koca and Saatçı, 2022; Bağatarhan et al., 2023), and few have compared their independent and serial effects within the same model. Therefore, drawing on the I-PACE model as the overarching theoretical framework, this study aims to explore how perceived parental phubbing affects adolescent Internet gaming disorder symptoms through the independent and serial mediating effects of fear of missing out and self-control, and to compare the differential effects of the two mediating pathways.

## 2 Participants and methods

### 2.1 Participants

Convenience sampling was used to select junior and senior high school students from six schools in Hebei Province as participants. A total of 5,700 questionnaires were collected. After invalid questionnaires (e.g., those with missing information) were excluded, 5,476 valid questionnaires remained. Among them, 2,756 participants were boys, 2,713 were girls, and 9 had missing gender information; 3,247 were junior high school students and 2,231 were senior high school students; 3,000 were urban students, 2,438 were rural students, and 40 had missing information; 857 were only children, 4,602 were non-only children, and 19 had missing information; 3,073 ranked in the upper half of academic performance, 2,265 ranked in the lower half, and 140 had missing information; 2,893 had relatively high family socioeconomic status, 2,405 had relatively low family socioeconomic status, and 180 had missing information.

### 2.2 Measures

#### 2.2.1 Internet Gaming Disorder Symptoms Scale

The scale was revised by Zhang et al. (2024) and includes six items, such as ‘Playing online games has become part of my daily routine.’ It uses a 5-point scoring system (1 = strongly inconsistent, 5 = strongly consistent). Higher scores indicate more severe Internet gaming disorder symptoms. The omega coefficient of the total scale was 0.89. The construct validity is satisfactory (*χ*^2^/df = 30.713, GFI = 0.987, CFI = 0.989, TLI = 0.976, RMSEA = 0.074, RMR = 0.027).

#### 2.2.2 Perceived Parental Phubbing Scale

This scale was adapted from the Perceived Parental Phubbing Scale revised by Ding et al. (2020). It consists of four items and uses a 5-point scoring system (1 = never, 5 = always) to measure adolescents’ perceived parental phubbing. Higher scores indicate a higher degree of perceived parental phubbing. The omega coefficient of the total scale was 0.75. The construct validity is satisfactory (χ^2^/df = 31.708, GFI = 0.994, CFI = 0.988, TLI = 0.963, RMSEA = 0.075, RMR = 0.022).

#### 2.2.3 Self-Control Scale

Developed by Partsch and Danner (2021), this scale contains four items, such as ‘I am good at resisting temptation.’ It uses a 5-point scoring system (1 = strongly inconsistent, 5 = strongly consistent), with higher scores indicating stronger self-control. The omega coefficient of the total scale was 0.83. The construct validity is satisfactory (χ2/df = 4.355, GFI = 1.000, CFI = 1.000, TLI = 0.998, RMSEA = 0.025, RMR = 0.005).

#### 2.2.4 Fear of Missing Out Scale

This scale was adapted from the Fear of Missing Out Scale developed by Przybylski et al. (2013). It consists of three items, such as ‘I worry that others have more meaningful experiences than I do.’ It uses a 5-point scoring system (1 = strongly inconsistent, 5 = strongly consistent), with higher scores indicating a higher level of fear of missing out. The omega coefficient of the total scale was 0.78.

### 2.3 Data analysis

SPSS 27.0 was used for descriptive statistics and correlation analysis, and PROCESS Model 6 was used to test the significance of mediation effects. Bootstrap resampling was performed 5,000 times to calculate bias-corrected 95% confidence intervals for testing the significance of mediation effects. If the confidence interval did not include 0, the mediation effect was considered significant (Hayes, 2017).

## 3 Results

### 3.1 Common-method bias

Harman’s single-factor test was used to examine common method bias (Podsakoff et al., 2003). The results showed that there were four factors with eigenvalues >1, and the variance explained by the first factor was 29.63%, below the critical value of 40%, indicating that common method bias was not serious.

### 3.2 Preliminary analyses

Pearson product–moment correlation analysis showed that perceived parental phubbing and fear of missing out were both significantly and positively correlated with Internet gaming disorder symptoms, whereas self-control was significantly and negatively correlated with Internet gaming disorder symptoms (Table 1).

**Table 1:** Descriptive statistics and correlations (*n* = 5,476).

Variable	M ± SD	1	2	3	4
1. Gender (1 = boys, 2 = girls)	1.59 ± 0.49				
2. Perceived parental phubbing	2.90 ± 1.07	−0.06**			
3. Fear of missing out	2.47 ± 1.03	−0.17**	0.23**		
4. Self-control	3.28 ± 0.86	0.07**	−0.19**	−0.26**	
5. Internet gaming disorder symptoms	2.11 ± 0.97	0.28**	0.20**	0.22**	−0.30**

**p < 0.01.

### 3.3 Mediation effect tests

**Figure 1:**
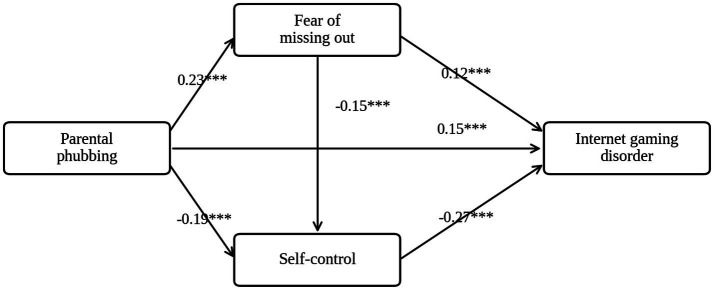
Serial mediation model. Unstandardized regression coefficients are shown. ***p < 0.001.

The results showed that perceived parental phubbing not only significantly and directly predicted adolescents’ Internet gaming disorder symptoms, but also indirectly increased the risk of Internet gaming disorder symptoms through the independent mediating effects of fear of missing out and self-control, as well as through the serial mediating pathway involving both variables. The mediating effect of fear of missing out (Path 1) accounted for 40.17% of the total indirect effect; the mediating effect of self-control (Path 2) accounted for 43.34% of the total indirect effect; and the serial mediating effect (Path 3) accounted for 16.49%. Tests of differences among the mediation effects showed that the difference between Path 1 and Path 2 was not significant, whereas both differed significantly from Path 3 (Figure 1; Tables 2, 3).

**Table 2:** Mediation effect tests.

Path	Effect	Boot SE	Boot CI lower	Boot CI upper	*p*
Path 1: Mediation via FoMO	0.04	0.00	0.03	0.05	<0.001
Path 2: Mediation via self-control	0.04	0.00	0.03	0.05	<0.001
Path 3: Serial mediation	0.02	0.00	0.01	0.02	<0.001
Total indirect effect	0.09	0.01	0.08	0.11	<0.001
Direct effect	0.15	0.02	0.11	0.18	<0.001
Total effect	0.24	0.02	0.21	0.27	<0.001

FoMO, fear of missing out; Boot, bootstrap; CI, confidence interval.

**Table 3:** Differences among mediation pathways.

Path contrast	Effect difference	95% CI	*p*
Path 1 vs. Path 2	−0.003	[−0.017, 0.011]	>0.05
Path 1 vs. Path 3	0.022	[0.012, 0.031]	<0.001
Path 2 vs. Path 3	0.025	[0.015, 0.034]	<0.001

## 4 Discussion

### 4.1 Association between perceived parental phubbing and Internet gaming disorder symptoms

The results showed that perceived parental phubbing positively predicted adolescents’ Internet gaming disorder symptoms, supporting Hypothesis 1 and aligning with previous findings (Wang et al., 2026). As a form of implicit emotional neglect, perceived parental phubbing may obstruct the satisfaction of adolescents’ normal attachment needs and violate their expectations for a parent–child relationship characterized by warm companionship and timely responsiveness (Yin et al., 2024). When real-life emotional support is insufficient and negative emotions are difficult to relieve, online games, with their high-intensity emotional arousal and social-compensatory functions, become an important medium through which adolescents alleviate psychological pain, escape real-life neglect, and fill emotional gaps (Wegmann et al., 2025). Therefore, perceived parental phubbing is significantly and positively correlated with adolescents’ Internet gaming disorder symptoms.

### 4.2 The mediating role of fear of missing out

The results confirmed that fear of missing out partially mediated the association between perceived parental phubbing and adolescent Internet gaming disorder symptoms, supporting Hypothesis 2, and aligning with previous findings (Yin et al., 2024). As a new form of neglectful behavior, perceived parental phubbing not only directly undermines the quality of parent–child interaction, leaving adolescents’ needs for belonging and emotional support chronically unmet, but also hinders the establishment of secure attachment relationships and intensifies adolescents’ fear of missing out (Geng et al., 2021; Wang et al., 2026). The social connection, achievement feedback, and reward system provided by online games (Pirrone et al., 2024) can rapidly fill real-life emotional gaps and temporarily alleviate unease and deprivation caused by anxiety. When individuals repeatedly turn to online games to avoid negative emotions, this behavior is likely to become solidified as an automatic coping pattern and eventually develop into Internet gaming disorder symptoms (Yin et al., 2024).

### 4.3 The mediating role of self-control

The results showed that self-control mediated the association between perceived parental phubbing and adolescent Internet gaming disorder symptoms, supporting Hypothesis 3, and aligning with previous findings (Li et al., 2026). The interpersonal stress and negative emotional experiences caused by perceived parental phubbing constitute a high-intensity source of ego depletion. To alleviate inner discomfort, adolescents must continuously mobilize limited psychological resources for emotion regulation and cognitive reconstruction (Jiang et al., 2023). When individuals’ psychological resources are chronically in a state where ‘consumption exceeds recovery,’ their self-control ability declines significantly (Baumeister et al., 2024). Individuals with lower levels of self-control have limited inhibitory control when facing the temptation of online games, and this is associated with a higher tendency toward Internet gaming disorder symptoms (Cui et al., 2024).

### 4.4 Serial mediating effects

The results showed that fear of missing out and self-control played a serial mediating role between perceived parental phubbing and adolescent Internet gaming disorder symptoms, supporting Hypothesis 4, and aligning with previous findings (Ding et al., 2026; Gan et al., 2024). Perceived parental phubbing not only damages emotional bonds between parents and children, but also makes adolescents develop strong fear of missing out because their need for belonging is blocked (Tong et al., 2024). A prolonged state of anxiety consumes individuals’ limited psychological resources and weakens their rational regulation and impulse-inhibition abilities (Ding et al., 2026). When self-control decreases, adolescents find it more difficult to resist the immediate rewards and emotional compensation brought by online games (Liu et al., 2025).

It is worth noting that in this study, the effects of the two independent mediation pathways through fear of missing out and self-control were both significantly larger than the serial mediating pathway. This indicates that two relatively independent and stronger channels may simultaneously exist between parental phubbing and adolescent Internet gaming disorder symptoms. One is the emotion-driven pathway, in which adolescents use online games to alleviate fear of missing out, lack of belonging, and social anxiety. The other is the executive-control pathway, in which adolescents with lower self-control have difficulty resisting immediate gaming rewards and controlling gaming duration. By comparison, the serial pathway must proceed through the sequential process of ‘increased fear of missing out → decreased self-control → increased tendency toward Internet gaming disorder symptoms,’ which includes more intermediate links and therefore has a relatively smaller effect. This result suggests that fear of missing out and self-control are not simply connected in a single sequential path, but may represent, respectively, emotional risk mechanisms and control-related risk mechanisms in the development of adolescent Internet gaming disorder symptoms. From the perspective of the family environment, this result extends the I-PACE model and reveals how insufficient parent–child interaction can simultaneously influence adolescents’ emotional-motivational system and executive-control system, providing empirical support for understanding the multi-pathway formation mechanism of adolescent Internet gaming disorder symptoms.

### 4.5 Implications and limitations

The integrated model constructed in this study quantitatively confirms that the emotion-driven pathway and the self-control impairment pathway are the core dominant mechanisms, while the serial pathway serves as a secondary supplementary mechanism, thereby refining the differentiated pathways through which the family environment influences adolescent gaming addiction. The findings suggest that parents should abandon the undesirable parenting state of being ‘physically present but digitally absent’ during parent–child companionship and should appropriately improve the quality of companionship and communication to promote more stable emotional responses. Second, digital literacy education may be combined with mental health education to guide students in understanding the reward mechanisms of online games and help them identify persistent online impulses caused by fear of missing out. Finally, the significant mediating role of self-control suggests that future intervention research may further explore whether time-management and delayed-gratification training can help improve adolescents’ gaming-related problems.

This study still has several limitations. First, it adopted a cross-sectional questionnaire design, making it difficult to fully reveal the dynamic processes through which the variables unfold over time. Therefore, caution is needed when interpreting the relationships among variables and proposing intervention suggestions. Future research may adopt longitudinal tracking designs, cross-lagged models, or experimental paradigms to examine the causal directions and temporal evolution of the variables more rigorously. Second, this study examined only mediation mechanisms and did not explore in depth the boundary-moderating roles of variables such as family environment and individual traits in this mediation model. Future research may further introduce moderating variables such as gender and parent–child relationship quality to explore the model’s boundaries of applicability and group differences. At the same time, the geographical scope of the sample in this study remains relatively limited. Future research may expand samples from different regions, school types, and family backgrounds to improve the generalizability of the findings. Finally, all variables in this study were measured through adolescents’ self-reports, which may involve common method bias. In addition, individuals’ psychological states and behavioral problems may also influence their subjective perceptions of parental behavior. For example, adolescents with higher fear of missing out, lower self-control, or more severe Internet gaming disorder symptoms may be more inclined to interpret and report their parents’ mobile-phone use behavior negatively, thereby introducing negative perception bias. Future studies may combine parental reports or objective behavioral indicators for multisource data collection to further improve the reliability of the findings.

## 5 Conclusion

Perceived parental phubbing is significantly and positively correlated with adolescent Internet gaming disorder symptoms, and the independent mediating effects and serial mediating effects of fear of missing out and self-control between perceived parental phubbing and Internet gaming disorder symptoms are significant. In summary, these findings suggest that efforts to prevent adolescent Internet gaming disorder symptoms in the digital age might benefit from attention to parents’ digital parenting behaviors, adolescents’ fear of missing out, and their self-control capacities.

## Data Availability

The raw data supporting the conclusions of this article will be made available by the authors, without undue reservation.
